# The structural integrity of human TFF1 under reducing conditions

**DOI:** 10.1016/j.redox.2025.103534

**Published:** 2025-02-05

**Authors:** Dilsah Nur Elmaci, Gene Hopping, Werner Hoffmann, Markus Muttenthaler, Matthias Stein

**Affiliations:** a Molecular Simulations and Design Group, Max Planck Institute for Dynamics of Complex Technical Systems, 39106 Magdeburg, Germany; b Institute for Molecular Bioscience, The University of Queensland, Brisbane 4072, Australia; c Institute for Molecular Biology and Medicinal Chemistry, Medical Faculty Otto von Guericke University, 39120 Magdeburg, Germany; d Institute of Biological Chemistry, University of Vienna, 1090 Vienna, Austria

**Keywords:** Trefoil factor family, Disulfide bond stability, Reductive stability, Molecular dynamics simulation

## Abstract

The trefoil factor family (TFF) comprises three secretory peptides (TFF1, TFF2, TFF3) that regulate diverse physiological processes to maintain gastrointestinal mucosal integrity and homeostasis. The TFF domain is stabilized by six conserved cysteine residues forming three intramolecular disulfide bonds. In this work, we investigated human TFF1 domain stability against increasing concentrations of the reducing agent tris(2-carboxyethyl)phosphine (TCEP). Experiments revealed high resistance of the disulfide bonds within the TFF1 domain to reduction compared to two reference peptides with similar three-disulfide frameworks, namely the bovine pancreatic trypsin inhibitor (BPTI) and the peptide drug linaclotide. Full reduction of TFF1 was only achieved with a large excess of TCEP (150-fold), and no partially reduced intermediates were observed, supporting a compact TFF1 domain. This TFF1 domain stability was supported by extensive all-atom molecular dynamics simulations for a total of 24 μs of all possible combinatorial states of disulfide bond reduction. Despite minor structural and conformational changes observed upon reduction, the domain substantially retained its overall compactness and solvent exposure when only one or two disulfide bonds were removed. The reduced cysteine residues did not undergo large structural rearrangements and remained buried. The loss of covalent disulfide bonds upon reduction was counterbalanced through persistent non-covalent interactions. These molecular simulations explained why TFF1 could not be partially reduced and alkylated during the experiments despite titrating different TCEP concentrations in the presence of alkylating agents. Our findings provide the first insights into the remarkable stability of the human TFF domain under reducing conditions, supporting its functional resilience upon expression and secretion throughout the human body.

## Highlights

• Human trefoil factor 1 (TTF1) protein is stabilized by three intramolecular disulfide bonds.
• Complete reduction of disulfide bonds of TFF1 occurs only in large excess of reducing agent.
• TFF1 shows enhanced stability compared to reference peptides BPTI and linaclotide.
• MD simulations reveal details of non-covalent stabilization across different redox states.

## Graphical abstract

**Figure:**
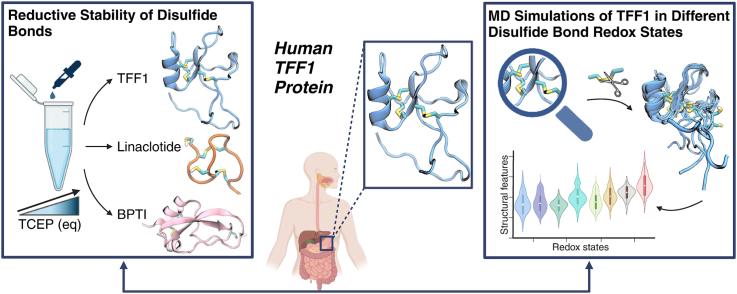

## 1 Introduction

Disulfide bonds are critical constituents in peptides and proteins, contributing to folding, structural stability, and functionality [[1], [2], [3]]. They stabilize the folded state by promoting local interactions, such as hydrophobic interactions, and enhance the structural integrity of peptides and proteins, thereby increasing their resistance to denaturation and proteolytic degradation under extracellular environmental conditions [1,4].

Disulfide bonds are susceptible to reduction by specific enzymes, including thioredoxin and glutaredoxin, as well as redox molecules, such as glutathione [5,6]. They can also be reduced using chemical reagents such as tris(2-carboxyethyl)phosphine (TCEP) or dithiothreitol (DTT) [2]. Upon reduction, structural and dynamic changes may occur, including destabilization of the folded state or of local regions near the disulfide bonds [7]. These changes may result in a reduction or complete loss of protein activity [8,9]. Reduction can also be a mechanism to enable further degradation by enzymes [6,10].

Many secretory proteins contain disulfide bonds, with most formed during folding in the endoplasmic reticulum [11,12]. The trefoil factor family (TFF) is a highly relevant example of such secretory peptides in humans, with three characteristic peptides (TFF1, TFF2, TFF3) produced by mucous epithelia. They are most abundantly secreted throughout different compartments of the gastrointestinal tract, where they have important functions in protecting the epithelium barrier and maintaining mucosal epithelial homeostasis [13,14]. Each member of the family has at least one highly conserved TFF domain containing six cysteine residues (C^I^(X)_9-10_C^II^(X)_9_C^III^(X)_4_C^IV^C^V^ (X)_9_C^VI^; C: cysteine, X: any amino acid), which stabilize the domain by forming three intramolecular disulfide bonds between Cys^I−V^, Cys^II−IV^_,_ and Cys^III−VI^, resulting in a three-looped fold reminiscent of a trefoil [13]. This three-dimensional structure is critical for structural stability, supporting TFF's functions in the diverse and harsh environments of the gastrointestinal tract [15], which include a wide range of pH levels and proteolytic enzymes [16]. The TFF domain demonstrates resistance to gastrointestinal proteases for TFF2 and TFF3 peptides [[17], [18], [19], [20], [21]], pointing to their sustained functionality within the gastrointestinal tract. For TFF3, the N- and C-termini are degraded under gastric and intestinal conditions, giving rise to a fully gut-stable and bioactive TFF3 metabolite that retains the TFF domain with protective flanking residues [22]. In line with this, the N- and C-terminal regions outside the TFF domain have been identified as targets for proteolytic degradation, as shown for TFF3 in saliva, as well as in bronchial and cervical secretions [[23], [24], [25]]. Disulfide bonds within both monomer and homodimer TFF3 domains were also observed to persist in the presence of reduced glutathione (10 equivalents, 100 μM, pH 7), with no disulfide bond reduction or scrambling detected [22]. Cysteine-to-serine mutations in the TFF3 domain resulted in a loss of proteolytic resistance and its capability of inducing cell migration [19].

**Fig. 1:**
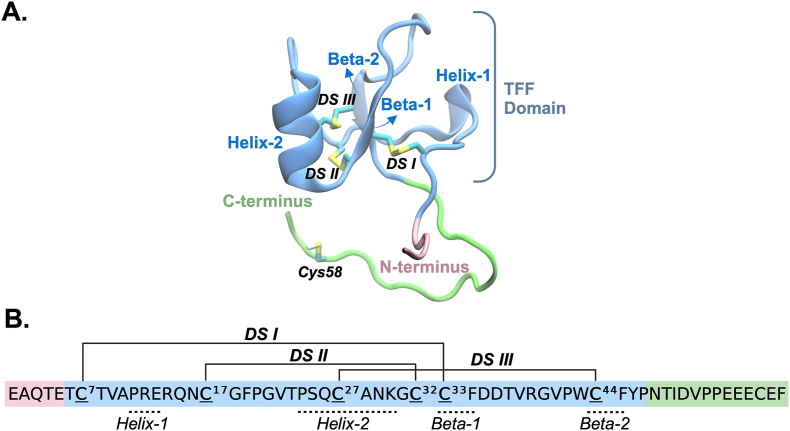
Structure of monomeric human TFF1 with its characteristic disulfide bonds DSI, DSII, and DSIII between residues Cys^7^-Cys^33^, Cys^17^-Cys^32^, and Cys^27^-Cys^44^, respectively. The C-terminal 7^th^ unpaired cysteine residue Cys^58^ is also indicated. (A) Cartoon representation of TFF1 with the N- and C-termini in pink and green, respectively. The TFF1 domain is highlighted in blue, and the three disulfide bonds are shown in yellow color in a licorice representation. (B) Amino acid sequence of TFF1 with labeled intramolecular disulfide bonds.

Among the three TFF members (TFFs), TFF1 comprises 60 amino acids and is primarily expressed by gastric surface mucous cells. Additionally, TFF1 is found in body fluids, including tears, saliva, urine, and breast milk [14,26]. In TFF1, the TFF domain extends from residues 6–47, and the three intramolecular disulfide bonds are formed between Cys^7^-Cys^33^, Cys^17^-Cys^32^, and Cys^27^-Cys^44^(Fig. 1). A C-terminal 7^th^ cysteine residue (Cys^58^ ) is located outside the TFF1 domain, flanked by four glutamic acidic residues (E^55^EEC^58^E^59^) [13,14]. A perturbed pKa of that cysteine residue may be why TFF1 retains its monomeric structure with this unpaired free cysteine residue, as observed in human, mouse, and *Xenopus laevis* (ortholog xP1) [14,27].

Considering TFF1's physiological roles, it may be part of the inner insoluble gastric mucus barrier since considerable amounts of TFF1 bound to GKN2 are present in a hardly soluble form [27]. Human TFF1 was recently synthesized using a two-fragment ligation strategy, and it appears to carry out its protective actions in the gut through lectin-like mucin cross-linking [28]. Homodimeric TFF1 binds as a lectin to the core oligosaccharide of wild-type *Helicobacter pylori* as well as to the gastric mucin MUC6 [27,29]. This lectin activity may also be responsible for the role of TFF1 as an antral tumor suppressor by low-affinity binding to the carbohydrate moiety of a transmembrane glycoprotein [30]. A growing number of glycoproteins are claimed to bind TFFs [30,31]; however, the specific binding of TFF1 to these glycoproteins has yet to be demonstrated.

Although a crystal structure of the domain-containing TFF1 has been resolved [32], this static snapshot does not reveal the dynamics and roles of the individual disulfide bonds in preserving TFF1's structural integrity and their susceptibility to reduction. In this regard, molecular dynamics (MD) simulations can explore conformational changes of such biomolecular systems in response to particular mutational or redox perturbations [33]. Several computational studies have investigated structural changes in peptides and proteins upon breaking their disulfide bonds [[34], [35], [36]]. For instance, for human serum albumin, short 70 ns of MD simulations were used to show that disulfide bonds are essential to maintain its global shape; their removal affects binding sites and aggregation propensity of the protein [34]. In addition, the disulfide bond breakage pathway and reduction effects on the dimerization were investigated for human α-defensin type 5 and β-defensin type 3 upon removal of all disulfide bonds. This process was driven by an increase in entropy for reduced α-defensin type 5 and solvent-mediated for β-defensin type 3 protein [35]. In another example, all-atom MD simulations of sequential disulfide bond removal in the dimeric *Solanum tuberosum* plant-specific insert protein showed that its disulfide bonds were not necessarily critical for maintaining its tertiary structure. An extensive network of hydrogen bonds within the monomers and hydrophobic interactions at the dimer interface stabilized the protein's local secondary structures and tertiary fold regardless of the presence of disulfide bonds. The overall structural integrity of this defensive protein was suggested to be critical to maintaining its antimicrobial action even at low pH [36].

Besides computational studies, experimental investigations have shed light on the role of disulfide bonds in protein stability and activity, often by treating them with reducing agents [37,38]. As an example, the effects of disulfide bond cleavage on protein aggregation were explored using two model proteins, lysozyme and bovine serum albumin (BSA) [39]. These proteins were treated separately with DTT and TCEP at pH 7.2 and 37 °C. Disulfide bond reduction in both proteins increased their susceptibility to forming amorphous aggregates with distinct morphologies. Additionally, incomplete disulfide reduction resulted in the formation of scrambled disulfide bonds in both proteins, suggesting an alternative pathway for protein aggregation, favoring the formation of amorphous aggregates over amyloid-like structures. Another study highlighted the significance of disulfide bond presence in the receptor-binding domain (RBD) of the SARS-CoV-2 Spike protein [40]. *In vitro* reduction of these bonds with DTT and TCEP altered RBD's secondary structure and lowered its melting temperature. This destabilization led to increased structural flexibility and partial unfolding at physiological temperature, which decreased the binding affinity of the RBD to Spike cell receptor ACE2 by two orders of magnitude. These examples demonstrated how experimental and computational methods can provide a holistic understanding of how disulfide bonds govern protein structure, function, and dynamics, highlighting their critical roles in diverse biological contexts.

In this study, we investigated the reductive stability of the disulfide bonds in human TFF1 and assessed their impact on the structural integrity and dynamics of the TFF1 domain using both experimental and computational approaches. TFF1 was incubated with various concentrations of reducing TCEP, and the disulfide bond reduction was analyzed by analytical reversed-phase high-performance liquid chromatography (RP-HPLC) and mass spectrometry (MS). These experiments were complemented by, in total, 24 μs of all-atom MD simulations of TFF1 with its disulfide bonds reduced in all possible permutations to elucidate structural changes across different reduction states. The observed stability of the TFF1 domain under reducing experimental conditions could be explained by minor global and local changes seen in the MD trajectories, particularly by its remarkable compactness and conserved solvent accessibility. This joint experimental and computational approach explains and supports the intriguing integrity and stability of TFF1 within a reducing environment.

## 2 Materials and methods

### 2.1 Materials

With noted exceptions, all Fmoc-protected amino acids were from Iris Biotech GmbH (Marktredwitz, Germany). Oxyma-Pure, Fmoc-Asp(OMpe)-OH, Fmoc-His(Boc)-OH, Fmoc-Tyr(OtBu)-Wang and Fmoc-Phe-Wang polystyrene resins (0.3 meq/g) were from CEM Corporation (Matthews, NC, USA). 2-Iodoacetmaide (IAM) and all other chemicals were from Merck Life Science (Bayswater, Australia), except for tris(2-carboxyethyl)phosphine (TCEP), which was purchased from FluoroChem (Hadfield, United Kingdom). Bovine pancreatic trypsin inhibitor (BPTI) was supplied by ProSpec-Tany TechnoGene Ltd (Ness-Ziona, Israel) and was further purified by RP-HPLC prior to use.

### 2.2 Solid-phase peptide synthesis

TFF1 and the three-disulfide bond reference peptide drug linaclotide were synthesized on a CEM Liberty Prime Microwave Peptide Synthesizer and subsequently cleaved, folded, and purified using methods previously described [22,41].

### 2.3 RP-HPLC and LC-MS

Peptides were purified by preparative RP-HPLC using a C_5_ column (Phenomenex Luna; 10 μm, 21.2 mm ID × 250 mm; flow rate 20 mL/min) on a Shimadzu LC-20A system running a linear gradient of solvents A (0.1 % TFA (aq) and B (90 % acetonitrile (aq)/0.1 % TFA). Fractions were analyzed by liquid chromatography-mass spectrometry (LC-MS), and those containing the desired peptide and >95 % purity were pooled and lyophilized. LC-MS analyses were performed on a C_18_ column (Agilent Eclipse Plus; 5 mm, 2.1 mm ID x 50 mm; flow rate 0.25 mL/min), using a QSTAR Elite electrospray ionization quadrupole time-of-flight (ESI QqTOF) mass spectrometer (SCIEX) linked to an upstream Shimadzu LC-20A HPLC system. HPLC solvents were the same as described previously, substituting formic acid for TFA. Peptides were separated on a gradient of 2–60 % B over 10 min and analyzed over a mass range of 200–2000 amu.

### 2.4 Reduction and alkylation

Peptides (TFF1, linaclotide, or BPTI) were dissolved to a concentration of 150 μM in 100 mM tris.HCl, pH 7.4. TCEP and IAM were prepared as separate 0.5 M solutions in 100 mM tris.HCl and the pH of all solutions were adjusted with concentrated KOH to pH 7.4. 25 μL of peptide (3.75 nmol) were treated with 0.5 M TCEP (0–150 molar equivalents per peptide). The samples were incubated at room temperature (25 °C) for 30 min, whereby they were treated with 5 μL (2.5 μmol; 100 eq/Cys) IAM for 5 min, followed by quenching with 5 μL TFA to reduce the pH to 2–3. Samples were then analyzed by LC-MS.

### 2.5 System preparation for molecular dynamics simulations

The crystal structure of human TFF1 (PDB ID: 6V1D, chain A) [32] was retrieved from the Protein Data Bank (PDB) [42,43]. Native TFF1 was reconstructed by substituting the N-terminal pyroglutamic acid with native Glu and reverting Leu^51^ to Asp^51^ to recover the native sequence (UniProt ID: P04155). The missing residues Glu^1^-Ala^2^ and final C-terminal residues (V^52^PPEEECEF^60^) were modelled using MODELLER and selected based on the DOPE-HR score [44,45]. Protonation states of titratable residues were assigned at pH 7.4 using the ProteinPrep module of Maestro [46]. In the TFF1 crystal structure, disulfide bonds between Cys^7^-Cys^33^, Cys^17^-Cys^32^, and Cys^27^-Cys^44^ are present. CHARMM-GUI was used to prepare the simulation files [[47], [48], [49]]. The systems were solvated using the TIP3P water model [50], and the solvated systems were subsequently neutralized and given a physiological salt concentration of 0.15 M NaCl. The CHARMM36m force field was utilized [51]. Therefore, a total of 8 different systems were prepared, representing the all-intact (AI), mono- (R1-3), di- (R4-6), and all-reduced (AR) disulfide bonds of TFF1 (Table 1).

**Table 1:** Investigated redox states of TFF1. “I” denotes an intact, whereas “R” denotes a reduced disulfide bond.

	Disulfide Bonds				
Redox state	Cys^7^-Cys^33^	Cys^17^-Cys^32^	Cys^27^-Cys^44^	Number of replicates x simulation time	Total simulation time
**AI**	I	I	I	3 x 1 μs	3 μs
**R1**	R	I	I	3 x 1 μs	3 μs
**R2**	I	R	I	3 x 1 μs	3 μs
**R3**	I	I	R	3 x 1 μs	3 μs
**R4**	R	R	I	3 x 1 μs	3 μs
**R5**	I	R	R	3 x 1 μs	3 μs
**R6**	R	I	R	3 x 1 μs	3 μs
**AR**	R	R	R	3 x 1 μs	3 μs

### 2.6 Details of molecular dynamics simulations

All simulations were carried out using OpenMM, a high-performance toolkit for molecular simulations [52]. Long-range electrostatic interactions were calculated with the particle mesh Ewald algorithm [53,54], while non-bonded interactions were computed using a cut-off distance of 1.2 nm along with a switch distance of 1.0 nm. The systems were minimized for 5000 steps. Afterwards, they were equilibrated in an ensemble with constant number of particles (N), volume (V), and temperature (T; NVT) for 0.1 ns and subsequently in an ensemble with constant number of particles, pressure (P), and temperature (NPT) for another 0.1 ns. During the equilibration steps, the backbone heavy atoms were restrained by a harmonic potential of 500 kcal/mol nm^2^. The temperature of the systems was maintained at 310.15 K using a Langevin integrator with a 1 ps^−1^ friction coefficient [55]. The time-step was 2 fs. In the NPT equilibration, a Monte-Carlo barostat was also applied to keep the pressure constant at 1 bar, which was coupled every 25 integration steps [56]. For the production run, the restrained forces were released, and all simulations were run in the NPT ensemble. Every system was simulated for three replicates of 1 μs each, reaching 24 μs in total simulation time. Simulations of each replicate were started with different initial velocities assigned according to a Maxwell-Boltzmann distribution.

### 2.7 Trajectory analysis

Trajectories were visualized and scrutinized using Visual Molecular Dynamics (VMD) software [57]. Some trajectory analyses were performed with different built-in packages of GROMACS [58]. The entire trajectory of each replicate was used for the analyses unless otherwise specified. Images were rendered via the Tachyon ray tracing in VMD [59]. Data visualization was carried out using the Seaborn and Matplotlib libraries [60,61].

#### 2.7.1 Structural analysis

The root-mean-square deviation (RMSD) of backbone atoms of the TFF1 domain was calculated by aligning the trajectories to the domain of the starting structure of each redox state using *gmx rms*. To investigate the local dynamic properties of the domain, the root-mean-square-fluctuations (RMSF) of the backbone atoms of the TFF domain were calculated using *gmx rmsf*. The solvent-accessible surface area (SASA) of individual residues in the TFF domain was calculated via *gmx sasa* using a probe radius of 0.14 nm. To determine the relative solvent accessibility (RSA), the calculated SASA values were normalized to the largest possible SASA of a given residue [62]. The radius of gyration (RoG) was calculated using *gmx gyrate* to assess the compactness of the domain in the presence and/or absence of disulfide pairs.

#### 2.7.2 Monitoring of cysteine S_γ_-S_γ_ distances

The distances between the cysteine sulfur atoms of the disulfide bonds (i.e., Cys^7^-S_γ_···S_γ_-Cys^33^, Cys^17^-S_γ_···S_γ_-Cys^32^, and Cys^27^-S_γ_···S_γ_-Cys^44^) were calculated with *gmx distance* for each simulation system. These interatomic distances were converted into probability distributions using the kernel density estimation with the default settings of the Seaborn library [60].

#### 2.7.3 Non-covalent intramolecular stabilization

The number of hydrogen bonds within the domain and their occupancies were computed with the HBonds plugin of the VMD [57], based on a donor-acceptor distance of <3.50 Å and a donor-hydrogen-acceptor angle of >110°. The number of hydrophobic interactions was determined by generating a minimum distance contact map with a cutoff of 4.5 Å, using only the sidechain heavy atoms of hydrophobic amino acids. For salt bridges, the distance between the center of mass (CoM) of the oxygen atoms in the acidic sidechains and the CoM of the nitrogen atoms in the basic sidechains was measured using the Salt Bridges plugin of VMD [57]. A distance of <5.5 Å was used as the threshold for a salt bridge interaction.

#### 2.7.4 Changes in secondary structural elements upon reduction

Dynamic changes in secondary structures of TFF1 were investigated using the Timeline plugin of VMD [57,63]. Residues forming secondary structural elements within the TFF domain (i.e., helix-1, helix-2, beta-1, and beta-2 in Fig. 1) were identified based on the crystal structure (PDB ID: 6V1D). The propensity of each residue to adopt specific structural types (i.e., alpha helix, 3–10 helix, pi-helix, extended configuration, isolated bridge, turn, and coil) was quantified by counting the structural assignments throughout the entire trajectory. For each secondary structural element, the frequency of different structural types was calculated by summing the assignments for all residues constituting the element across the trajectory and averaging these values over three replicates for each redox state. These resulting averages were then normalized by dividing by the total number of data points and multiplying by 100 to represent the secondary structure frequency.

## 3 Results and discussion

### 3.1 Reductive stability of the disulfide bonds within the TFF1 domain

**Fig. 2:**
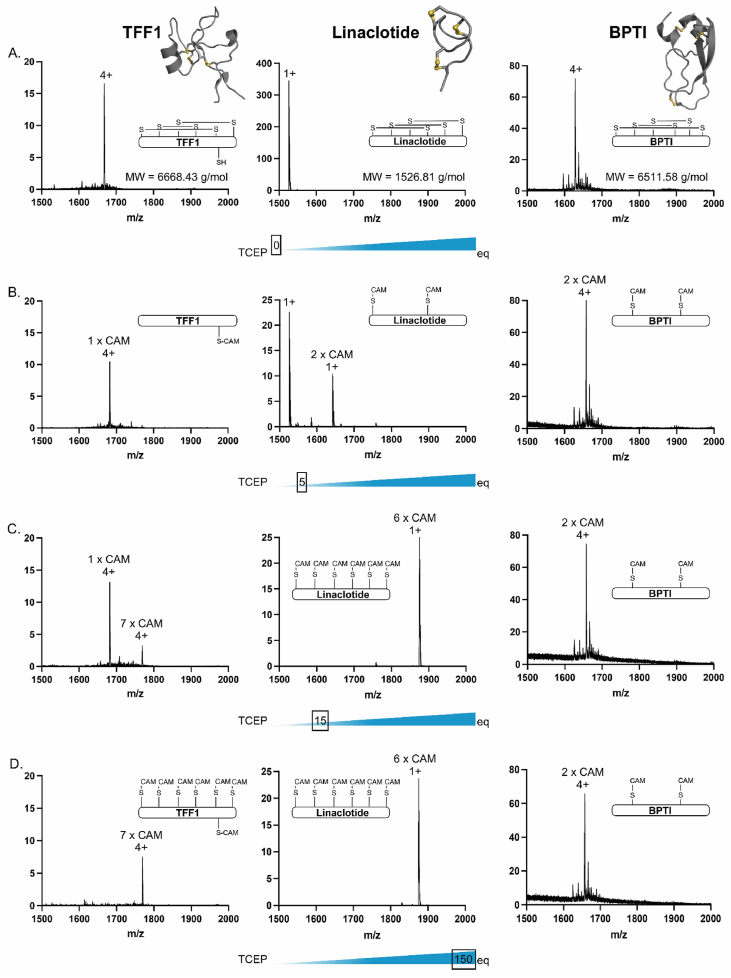
Reductive stability study of the TFF1 domain disulfide bonds and three-disulfide-bond-containing reference peptides, linaclotide and BPTI. Folded TFF1, linaclotide, and BPTI were individually exposed to increasing amounts of reducing agent TCEP (depicted in a blue slider underneath each set of panels) for 30 min at room temperature (25 °C), followed by the addition of iodoacetamide (IAM, 100-fold per Cys) and mass spectrometry analysis. The charge state of each ion species and the number of cysteine carboxyamidomethylations (CAM) resulting from reductive alkylation are shown above each peak (the CAM positions do not depict which exact Cys is being alkylated; they just indicate the number of alkylations observed). (A) Mass spectra of the starting materials TFF1, linaclotide, and BPTI prior to reduction. (B) Treatment of TFF1 with 5 molar equivalents of TCEP followed by the addition of excess iodoacetamide resulted in no reductive alkylation of the TFF1 domain disulfide bonds, only alkylation of the 7^th^ free cysteine (Cys^58^) outside the TFF1 domain. Treatment of linaclotide and BPTI under the same conditions resulted in partial reduction and alkylation of one disulfide bond for linaclotide, and complete reduction and alkylation of one disulfide bond for BPTI. (C) Increasing the TCEP concentration to 15 equivalents resulted in the appearance of a small fully reduced and alkylated TFF1 species in addition to the still intact TFF1 domain. The same conditions resulted in a complete reduction of all disulfide bonds in linaclotide but no further reduction of the remaining two disulfide bonds of BPTI. (D) Titration to 150 equivalents of TCEP was necessary to fully reduce and alkylate all disulfide bonds of TFF1, which also fully reduced and alkylated linaclotide but had no further effects on the remaining two buried disulfide bonds of BPTI.

To experimentally assess the stability of the three disulfide bonds of the TFF1 domain against reduction, we treated folded TFF1 with increasing concentrations of the reducing agent TCEP (0–150 equivalents per TFF1 molecule). We included linaclotide and bovine pancreatic trypsin inhibitor (BPTI) as reference compounds and compared them with TFF1 under the same conditions (Fig. 2(A)). Linaclotide is a 14-residue peptide drug administered orally for the treatment of irritable bowel syndrome with constipation and is characterized by three disulfide bonds in a Cys^I−IV^, Cys^II−V^, and Cys^III−VI^ connectivity [64,65]. Its disulfide bonds stabilize three β-turns and provide enhanced stability to linaclotide to exert its activity in the harsh gastrointestinal environment [[65], [66], [67]]. BPTI, on the other hand, is a larger 58-residue peptide with an alpha/beta fold constrained by three disulfide bonds in a Cys^I−VI^, Cys^II−IV^, and Cys^III−V^ connectivity [68,69]. It is a well-studied model peptide for folding and stability studies with a well-defined secondary structure and a hydrophobic core [68,70], making it a similar-sized reference compound for our studies.

Reduction for 30 min with 5 equivalents of TCEP prior to the addition of excess (600-fold) of the alkylating reagent IAM at room temperature showed no discernible reduction of the TFF1 domain; only the 7^th^ free cysteine (Cys^58^) outside the TFF1 domain was alkylated as expected (Fig. 2(B)). Under the same conditions, partial reduction and alkylation of a single disulfide bond were observed for linaclotide, and complete reduction and alkylation of a single disulfide bond was observed for BPTI (Fig. 2(B)). The absence of partially reduced and alkylated species in the TFF1 domain, unlike in linaclotide and BPTI, suggests that the TFF1 domain exhibits greater stability to reduction. Increasing the TCEP concentration to 15 equivalents resulted in two species in the TFF1 sample: the previously observed mono-alkylated species (7^th^ cysteine) and only a minor species corresponding to the fully reduced and alkylated peptide (Fig. 2(C)). Under the same conditions, linaclotide was fully reduced and alkylated, whereas BPTI showed no further reduction (Fig. 2(C)). TFF1 was then exposed to increasing concentrations of TCEP; 150 equivalents for 30 min at room temperature was necessary to observe complete reduction and alkylation of TFF1 (Fig. 2(D)). Linaclotide was again fully reduced and alkylated under these conditions. No further reduction of BPTI was observed even under these conditions (Fig. 2(D)), indicating that the final two disulfide bonds that are buried in BPTI's core remain resistant to reduction despite the early loss of the more accessible Cys^II−IV^ disulfide bond. Despite exhaustive trialing of different reducing conditions and temperatures, we could not reveal which TFF1 disulfide bond gets broken first. Taken together, the TFF1 domain was more resistant to reduction than the three-disulfide bond reference frameworks of linaclotide and BPTI.

### 3.2 Structural dynamics of TFF1: from oxidized to reduced states

#### 3.2.1 Global changes in the TFF1 domain

**Fig. 3:**
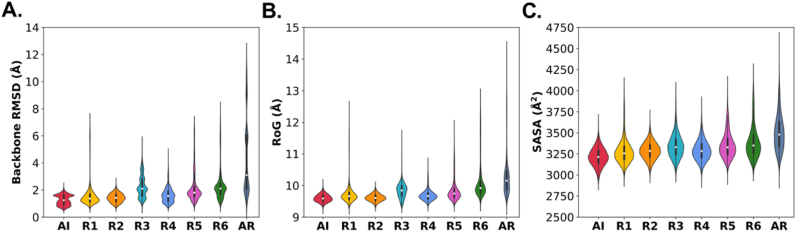
Changes in global properties of the TFF1 domain in different redox states. Violin plots depict the kernel density estimation of (A) backbone root-mean-square deviation (RMSD), (B) radius of gyration (RoG), and (C) solvent-accessible surface area (SASA) distributions. The length of embedded box plots represents the interquartile range with 50 % of the data and the white circle the median.

Deviations of the TFF1 domain from its crystal structure upon disulfide bond reduction were studied to provide more insights into the stability of the trefoil fold. Backbone RMSD profiles were calculated for each state (Fig. 3(A) and see Fig. S1(A) for timeline data). With all disulfide bonds intact (AI), the TFF1 domain was remarkably stable, as indicated by low RMSD values and a narrow RMSD distribution. Individual removal of DSI (R1) generally led to small deviations in domain stability when the entire duration of trajectories was considered. However, towards the end of one R1 trajectory, RMSD values reached up to 7 Å, indicating notable changes in the domain structure (see the last 150 ns of the R1 trajectory in Fig. S1(A)). Visual inspection of these frames revealed that this increase originated from a pronounced moving of the N-terminus away from the domain. The time evolution of these changes upon DSI reduction is detailed in Fig. S2 (see TFF1 domain colored in light blue in the second replicate of the R1 state). The reduction of the DSII bond alone did not result in significant structural changes. This suggests additional domain stabilization by non-covalent interactions in the vicinity of DSII or between the residues Cys^17^ and Cys^32^ to compensate for the loss of the DSII bond. In contrast, DSIII removal (in R3) led to increased RMSD values, indicating substantial deviations from the crystal structure, particularly arising from the destabilization of helix-2 (Fig. S2 and Fig. 7). These findings indicate that DSIII is crucial in maintaining domain stability as it connects helix-2 and beta-2 (Fig. 1). Simultaneous removal of the DSI and DSII bonds (in R4) led to only a minor increase in RMSD, similar to the R2 state. On the other hand, the reduction of DSIII in combination with either DSI (R6) or DSII (R5) led to the sampling of relatively larger RMSDs, as also seen for the R1 and R3 states. In the all-reduced state (AR), structural deviations from the crystal domain structure became more pronounced (Fig. S2), as observed by a broader RMSD distribution, due to the absence of stabilizing effect of disulfide bonds.

As a measure of domain compactness, the radius of gyration (RoG) was calculated for all reduction states of disulfide bonds (Fig. 3(B), Fig. S1(B) for timeline data). Based on our MD simulations, the RoG analysis showed a similar pattern to the RMSD. However, the RoG distributions in the R3 and AR states were relatively narrower than those of RMSD, indicating that structural alterations in the domain upon reduction had relatively minimal impact on its overall compactness. The reduction of all disulfide bonds in the AR state led to decreased compactness of the TFF domain, indicated by increased RoG values.

The SASA of the TFF domain was calculated to assess the degree of domain exposure to the surrounding solvent molecules (Fig. 3(C) and Fig. S1(C) for timeline data). Analysis of the SASA values followed a trend similar to RoG, showing that the domain surface largely conserved its solvent exposure across different reduction states. This indicates an absence of significant structural re-orientations in the TFF1 domain upon reduction. Despite conformational changes in the domain induced by reductions, the domain could retain its overall integrity and interaction with the surrounding environment.

### 3.3 Changes in S_γ_-S_γ_ distances upon reduction

**Fig. 4:**
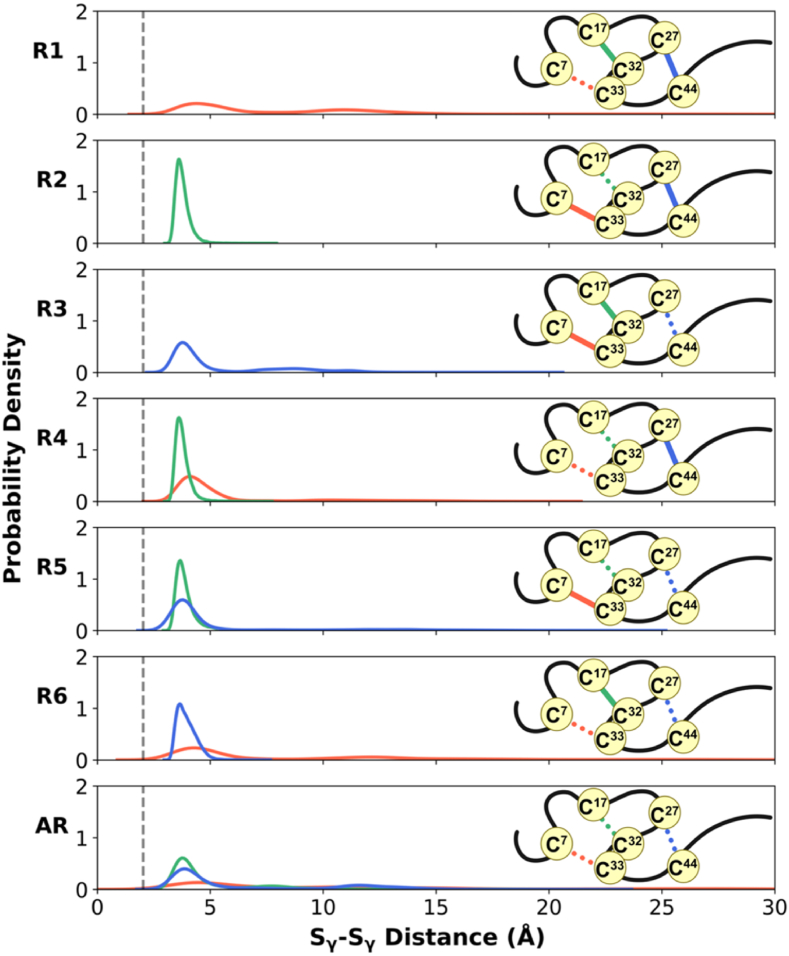
Probability density distributions of distances upon the sequential reduction of disulfide bonds, Cys^7^-S_γ_···S_γ_-Cys^33^ (red), Cys^17^-S_γ_···S_γ_-Cys^32^ (green), and Cys^27^-S_γ_···S_γ_-Cys^44^ (blue) in TFF1. In each panel, the reduced disulfide bonds are represented by dashed lines, whereas intact bonds are depicted as solid lines. The vertical grey dashed line denotes the S_γ_-S_γ_ distance in the crystal structure.

In the crystal structure of TFF1, the cystine sulfur distances are 2.03 Å, characteristic of a covalent S_γ_-S_γ_ bond. For each redox state, the S_γ_-S_γ_ distances of the reduced cysteine residues were monitored throughout the entire simulation time (Fig. S3). Redox state-dependent changes in S_γ_-S_γ_ distances were then analyzed using probability density distributions of these distances. Upon disulfide bond reduction, two typical distributions were observed: a distinct peak between 2.7 Å and 5 Å in S_γ_-S_γ_ distance and a broader distribution extending beyond 10 Å (Fig. 4).

The removal of DSI led to a very broad distribution of S_γ_-S_γ_ distances in general (panels R1, R4, and R6 in Fig. 4; red traces). It may be unexpected that the TFF domain maintained its structural stability upon DSI reduction for most of the simulation time, particularly in the R1 state (see backbone RMSD data in Fig. 3), despite the broader Cys^7^-S_γ_···S_γ_-Cys^33^ distance distribution. As shown in the 2D scatter plot of the Cys^7^-S_γ_···S_γ_-Cys^33^ distance and backbone RMSD of the domain in R1 (Fig. S4(A)), the larger sulfur atom distances between Cys^7^ and Cys^33^, which correspond to the broad distribution centered around 12 Å in Fig. 4, resulted in only minor structural changes in the domain, with low backbone RMSD values (∼1–2 Å). A significant change in the domain structure was observed only when the N-terminus of the domain moved substantially away due to reduction, causing the S_γ_-S_γ_ distance between Cys^7^ and Cys^33^ to exceed 20 Å. Since Cys^7^ is located near the flexible N-terminus and does not form persistent non-covalent interactions with other residues (Fig. S8), the occurrence of large distances between the sulfur atoms of Cys^7^ and Cys^33^ upon reduction is plausible. This makes the DSI bond susceptible to initial reduction. By contrast, when DSII was reduced, either alone or in combination with other disulfide bonds, its Cys^17^-S_γ_···S_γ_-Cys^32^distances remained remarkably stable at ∼3.6 Å (panels R2 and R4-5 in Fig. 4; green traces). The conservation of short distances might explain the notable structural stability and integrity of the TFF domain in the R2 state (Fig. 3 for RMSD, RoG, and SASA). Upon breaking DSIII (panels R3 and R5-6 in Fig. 4; blue traces), relatively larger Cys^27^-S_γ_···S_γ_-Cys^44^ distances were sampled. The 2D scatter plot of the Cys^27^-S_γ_···S_γ_-Cys^44^ distance and backbone RMSD in R3 (Fig. S4(B)) indicated that an increased S_γ_-S_γ_ distance between Cys^27^ and Cys^44^ in R3 led to slightly more pronounced structural changes in the domain upon reduction. Since DSIII links secondary structural elements within the domain and is also located at the core of the TFF1 domain, its reduction has a major impact on the domain structure, thereby significantly affecting its conformational dynamics. Without any covalent S_γ_-S_γ_ bond in the all-reduced state (AR), the distances between all sulfur atoms increased and resulted in broader distributions.

It might be expected that the TFF1 domain's adaption to disulfide bond removal would be a slow process; however, Fig. S3 shows that the S_γ_-S_γ_ distances of some reduced cysteine residues initially increased but then decreased shortly thereafter, reaching short distances. Therefore, no significant changes were observed in the S_γ_-S_γ_ distance distribution profiles when comparing the entire MD trajectories to only the last 250 ns of each replicate (Fig. S5).

### 3.4 Local structural perturbations upon reduction

**Fig. 5:**
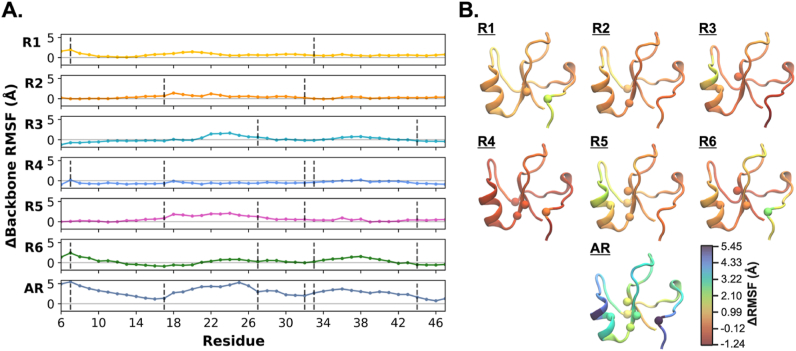
Changes in local residual fluctuations in the TFF domain upon reduction. A) Changes in backbone root-mean-square fluctuations (ΔRMSF) relative to the all-intact (AI) state. Vertical grey dashed lines indicate the positions of removed cysteine-cysteine bonds. B) Structural mapping of backbone RMSF changes to the crystal structure of TFF1. C_α_ atoms of reduced disulfide bonds are shown in van der Waals representation.

As a measure of residual flexibility and local changes in the TFF1 domain, relative changes in backbone RMSF per residue from the AI state (ΔRMSF) were calculated (Fig. 5). The backbone RMSF values of cysteine residues in the AI state, ranging from 2.17 Å to 3.44 Å, were used as a reference. When DSI between Cys^7^ and Cys^33^ was reduced, residual fluctuations of only the N-terminal Cys^7^ and nearby residues increased noticeably (see R1 and R6), whereas the flexibility of the corresponding Cys^33^ remained almost unaffected and only became more flexible in the fully reduced AR state. When DSII between Cys^17^ and Cys^32^ was removed (R2) alone or in combination with another disulfide bond (particularly in R4 and R5), Cys^17^ and Cys^32^ did not exhibit significant local fluctuations. However, the surrounding loop region (residues 18–23) generally displayed a slight increase in flexibility. It was unexpected to see that the simultaneous reduction of DSI and DSII (in R4) diminished the overall flexibility of the domain (Fig. 3 above for the TFF domain and Fig. 5 for relative changes in RMSF per residue). This suggested that the domain could maintain its stability upon reduction, possibly through conserved and long-living non-covalent interactions. Reduction of DSIII between Cys^27^ and Cys^44^ (in R3 and R5-6 states) led to an increase in the flexibility of helical residues 24–30; however, Cys^44^ and its neighboring residues remained relatively stable. Only when all disulfide bonds were reduced (AR), a significant increase in structural flexibility for the entire TFF1 domain was observed. Differences in RMSF values were generally observable only for residues in close spatial vicinity of the broken bonds and thus only local in nature.

The relative solvent accessibility (RSA) was calculated as a probe for a structural reorientation and possibly solvent exposure of TFF1 residues (Fig. S6). In the AI state, all cysteine residues in the domain were completely buried (RSA values close to 0; Fig. S6 and Table S1). Only Cys^7^ (in DSI) had a larger RSA of 0.4, which indicated its solvent accessibility. This supports the hypothesis of DSI being a candidate for the first reduction event. Typical hydrophobic residues (e.g., Val^9^, Val^22^, Val^41^, and Phe^45^) were also completely buried and thus may contribute to the hydrophobic stabilization of the domain. However, Arg^14^, one of the most hydrophilic residues, also displayed a low degree of solvent accessibility with a low standard deviation. This can be associated with the persistent salt bridge formation of Arg^14^ with Asp^35^ (Fig. S7).

**Fig. 6:**
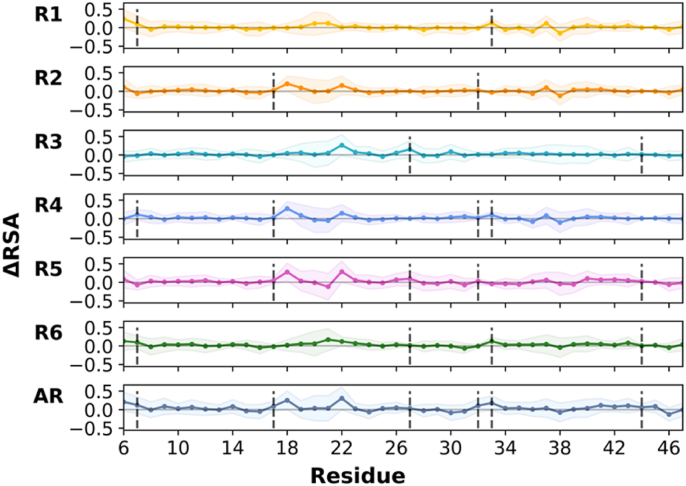
Changes in relative solvent accessibility (ΔRSA) per residue upon disulfide bond reduction from the all-intact state. Data points show the average ΔRSA values, and the shaded regions denote the standard deviation per residue. Vertical grey dashed lines indicate the positions of reduced cysteine residues.

Changes in RSA (ΔRSA) in all redox states became apparent from calculated differences in RSA per residue from the AI state (Fig. 6). In mono- and di-reduced states, breaking DSI (in R1, R4, and R6) led to increased solvent exposure of Cys^7^ and Cys^33^. However, breaking DSII (in R2 and R4-5) did not affect the residual solvent accessibility of reduced Cys^17^ and Cys^32^ residues. When DSIII was broken, Cys^27^ became only slightly more solvent-accessible, while Cys^44^ remained buried (in R3 and R5-6). In the case of the reduction of all disulfide bonds (AR), the exposure of cysteine residues to solvent slightly increased with higher standard deviations. The analysis of RSA changes per residue was thus consistent with the RMSF analysis of the entire TFF domain (Fig. 5).

These results revealed that mono- and di-reduction of disulfide bonds did not induce large structural reorientations or changes in the solvent exposure of free cysteine residues or the entire domain in general. This implies that some non-covalent intra-domain interactions may be conserved or even enhanced upon reduction, thereby maintaining the domain structure. This appears plausible since the domain is predominantly composed of hydrophobic residues, and cysteine residues are spatially close to each other. Considering the timescale of our simulations, the absence of notable changes in solvent exposure of both the entire domain (Fig. 3(C)) and individual free cysteine residues (Fig. 6) after mono- and di-reduction might explain the lack of experimentally observed partial alkylation for any of the TFF1 domain cysteine residues under mild reducing conditions. On the other hand, alkylation of the C-terminal Cys^58^ can easily be rationalized by its large solvent accessibility (RSA between 0.5 and 0.6) in all redox states (Fig. S6 and Table S1). As for the fully reduced TFF1, significantly larger changes in ΔRMSF (up to 5 Å per residue) were observed (Fig. 5(B) for structural mapping of residual fluctuations), showing that the domain backbone becomes more flexible in general. Even though the average RSA per residue in AR did not change significantly overall, the larger standard deviation suggested an enhanced structural fluxionality.

### 3.5 Changes in secondary structure upon TFF1 domain reduction

**Fig. 7:**
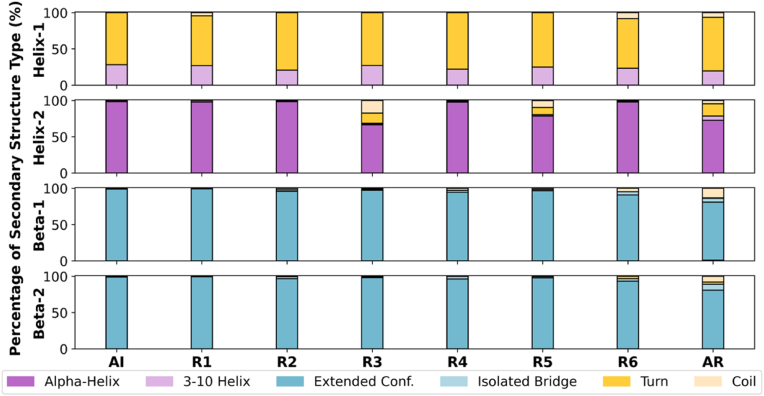
Changes in secondary structures of the TFF domain upon mono- (R1-R3), di- (R4-R6), and full reduction (AR). Propensities of secondary structural elements —helix-1, helix-2, beta-1, and beta-2— to adopt different structural types, including alpha-helix, 3–10 helix, extended configuration, isolated bridge, turn, and coil, are shown for each redox state.

Changes in the secondary structural elements of the TFF1 domain upon sequential removal of the disulfide bonds were investigated to explore their adaptation to different structural types (Fig. 7 and Table S2). In all redox states, helix-1 exhibited large structural variability and shuttled between a 3–10 helix and disordered elements (turn and coil). This kind of structural fluxionality was expected since helix-1 is a small one-turn helix composed of only three residues. Upon DSI reduction, no additional regular secondary structural changes were detected in the TFF1 domain. This could be anticipated, as Cys^7^ is in the disordered N-terminal tail, and Cys^33^ is part of beta-1, which is mainly stabilized by hydrogen bonds with beta-2. Breaking DSII between Cys^17^ and Cys^32^ (R2), even along with DSI (R4), did not affect the secondary structure of the TFF domain, as DSII connects unstructured loop regions in the oxidized state (AI). Helix-2 destabilization was likely to happen upon DSIII reduction due to the free Cys^27^ on this helix, as seen by a decrease in alpha-helix character and an increase in turn and coil elements in states R3, R5, and AR. In the case of simultaneous reduction of DSI and DSIII (R6), helix-2 maintained its secondary structure, suggesting that non-covalent interactions stabilizing helix-2 were preserved. The beta-sheet elements (beta-1 and beta-2) were generally very stable, showing almost no changes in the secondary structure for AI and R1-R5. However, when DSI and DSIII were reduced simultaneously (in R6 and AR), sheet elements beta-1 and beta-2 were slightly destabilized, probably due to an increase in the degree of freedom of Cys^33^, which is part of beta-1, and Cys^44^ as part of beta-2. Overall, significant changes in secondary elements towards structural disorder were only seen upon DSIII reduction or full reduction of the domain, but a complete TFF1 unfolding cannot be observed on the timescale of μs.

### 3.6 Non-covalent intradomain stabilization of the TFF1 domain

**Fig. 8:**
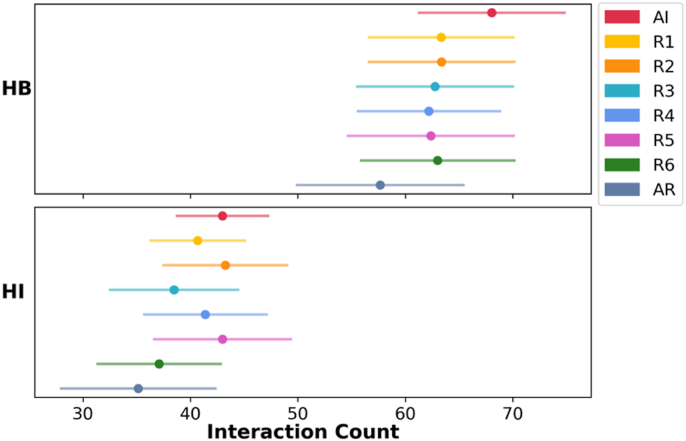
Intramolecular stabilization of the TFF domain: the number of hydrogen bonds (HB) and hydrophobic interactions (HI) in different redox states. The data presented are the average number of interactions per frame of the merged trajectories for each redox state, and error bars represent ± standard deviation.

Since the reduction of disulfide bonds led to a decrease in the number of covalent bonds stabilizing TFF1 but mostly without large structural reorientations, the number of non-covalent intra-domain interactions (hydrogen bonds and hydrophobic interactions) was investigated across all redox states (Fig. 8). Mono- and di-reduction led to only a slight decrease in the number of hydrogen bonds compared to the AI state. The largest decrease in the number of hydrogen bonds was seen in the AR state when all three disulfide bonds were reduced. Moreover, the number of hydrophobic interactions within the domain varied only slightly among the states, and the overall pattern agreed with the RoG profiles (Fig. 3(B)). The loss of hydrophobic interactions in the R3, R6, and AR states is also evident from the relatively higher RoG values observed in these states. In addition, only one salt bridge was observed between Arg^14^ and Asp^35^ in all redox states, which remained persistent for more than 50 % of the simulation time of all redox states (Fig. S7). Its presence was noticeably affected by the breaking of the DSI bond, particularly when the N-terminus of the domain became more distant from the domain. This strong interaction could also explain why the hydrophilic residue Arg^14^ stayed buried and did not become solvent-accessible upon reduction (Fig. 6).

A more detailed analysis of the hydrogen bonding stabilization for long-living pairwise interactions is presented in Fig. S8, revealing that cysteine residues in the TFF1 domain were not only participating in covalent disulfide bonds but also formed long-living hydrogen bonds with other cysteine residues, to which typically they do not form native disulfide bonds (i.e., Cys^17^-NH···O-Cys^44^, Cys^32^-NH···O-Cys^27^, and Cys^44^-H_α_···O-Cys^33^), or with other nearby residues (i.e., Cys^33^-NH···O-Phe^45^, Phe^45^-NH···O-Cys^33^, and Cys^27^-NH···O-Thr^23^). Due to the compact trefoil-like structure of the domain, cysteine residues could form persistent hydrogen bonds with residues that are sequentially distant but spatially close, independent of the reduction state. This also explains the only moderate elongation of S_γ_-S_γ_ distances between Cys^17^ and Cys^32^ upon DSII reduction. The persistent non-covalent interactions of cysteine residues highlighted the importance of their presence in maintaining the structural stability and integrity of the TFF domain.

In addition to cysteine residues, other residues in the TFF1 domain also contributed to the domain stabilization by establishing hydrogen bonds. As an example, the strong hydrogen bond between Asp^35^-NH···O-Trp^43^ was persistent even in the fully reduced state and thus contributed to the stabilization of nearby secondary structure elements beta-1 and beta-2. Hydrogen bonds between Phe^45^-HA···O-Gln^15^ and Tyr^46^-NH···O-Gln^15^ also supported the integrity of the domain core in all redox states. Hydrogen bonds forming helix-2 (e.g., Ala^28^-NH···O-Pro^24^, Asn^29^-NH···O-Ser^25^, Gly^31^-NH···O-Ala^28^, and Lys^30^-NH···O-Gln^26^) were strong and long-living in the presence of DSIII. However, when DSIII was reduced, a loss in these interactions could be observed, causing structural fluctuations in helix-2 (Fig. 7). To conclude, the intra-domain stabilization through long-living non-covalent interactions may rationalize how the TFF domain retains its structural integrity and compactness in mono- and di-reduced redox states.

## 4 Conclusion

TFF1 is a small secretory protein with a rigid TFF domain stabilized by three disulfide bonds. In this study, we experimentally demonstrated the pronounced resistance of the TFF1 domain's disulfide bonds against reduction and supported the observed findings with detailed MD studies. No partial reduction and alkylation were observed for the TFF1 domain, whereas reference peptides BPTI and linaclotide displayed partial or complete reduction and alkylation at the same molar equivalents of reducing agent TCEP. Full reduction and alkylation of all disulfide bonds in TFF1 was only possible in the presence of a large excess of TCEP (150-fold). We further investigated this remarkable stability through extensive MD simulations of all redox states of the disulfide bonds in the TFF1 domain. Upon mono- and di-reduction, minor global and local structural changes were observed within the TFF1 domain, particularly in the absence of DSI and DSIII. The reduction of DSIII mostly affected helix-2 stabilization, of which residue Cys^27^ is part of. Similarly, the simultaneous reduction of DSI and DSIII led to a disruption of sheets beta-1 and beta-2, as Cys^33^ (in DSI) and Cys^44^ (in DSIII) were part of these secondary structures. This underscored the critical role of DSI and DSIII in maintaining the TFF1 structure and fold. In the case of DSII reduction, the TFF1 domain remained remarkably stable due to enduring non-covalent interactions counteracting the loss of covalent bonding between Cys^17^ and Cys^32^. Despite minor changes in the structure and dynamics of TFF1 upon mono- and di-reduction, the overall compactness and solvent accessibility of the domain, including the mostly buried reduced cysteine residues, were hardly affected. This explained how the domain could not become easily accessible by alkylating agents. However, in the fully reduced state, the overall structural stability and domain compactness decreased, and the reduced cysteine residues became more solvent-accessible. The integrity of the domain structure in all mono- and di-reduced states could thus be rationalized by preserved long-living networks of hydrogen bonds and hydrophobic interactions within the domain, including contributions from cysteine residues and nearby additional residues. Even though a definite statement about the order of disulfide bond reductions could not be made from our findings, the initial breakage of DSI appears plausible considering its larger solvent accessibility, possibly followed by the reduction of the remaining disulfide bonds. However, computationally describing sequential disulfide bond reduction events requires a quantum chemical treatment, which will be covered in an upcoming publication.

In summary, our results provide several new insights into the remarkable stability of the TFF1 domain towards reduction, explain the absence of partial reduction and alkylation in our experiments, and underpin the TFF1 stability even under reducing conditions.

## CRediT authorship contribution statement

**Dilsah Nur Elmaci:** Writing – original draft, Visualization, Methodology, Investigation, Formal analysis. **Gene Hopping:** Writing – review & editing, Visualization, Investigation. **Werner Hoffmann:** Writing – review & editing, Supervision, Conceptualization. **Markus Muttenthaler:** Writing – review & editing, Supervision, Funding acquisition, Conceptualization. **Matthias Stein:** Writing – review & editing, Supervision, Resources, Methodology, Funding acquisition, Conceptualization.

## Declaration of competing interest

The authors declare that there is no financial or personal interest.

## Data Availability

Data will be made available on request.
