# Supplementary material for: The structural integrity of human TFF1 under reducing conditions
Source: Redox Biol. 2025 Feb 5;81:103534. doi: 10.1016/j.redox.2025.103534 (PMC11889601; doi:10.1016/j.redox.2025.103534)
Supplement: Multimedia component 1 [file mmc1.pdf]

Supporting Information

The structural integrity of human TFF1 under reducing conditions

Dilsah Nur Elmaci[a#], Gene Hopping[b#], Werner Hoffmann[c], Markus Muttenthaler[b,d*], Matthias Stein[a*]

[a] Max Planck Institute for Dynamics of Complex Technical Systems, 39106 Magdeburg, Germany.

[b] Institute for Molecular Bioscience, The University of Queensland, 4072 Brisbane, Australia.

[c] Institute for Molecular Biology and Medicinal Chemistry, Medical Faculty Otto von Guericke University, 39120 Magdeburg, Germany.

[d] Institute of Biological Chemistry, University of Vienna, 1090 Vienna, Austria.

[#] Contributed equally.

* Corresponding Authors:

Matthias Stein: matthias.stein@mpi-magdeburg.mpg.de

Markus Muttenthaler: m.muttenthaler@uq.edu.au

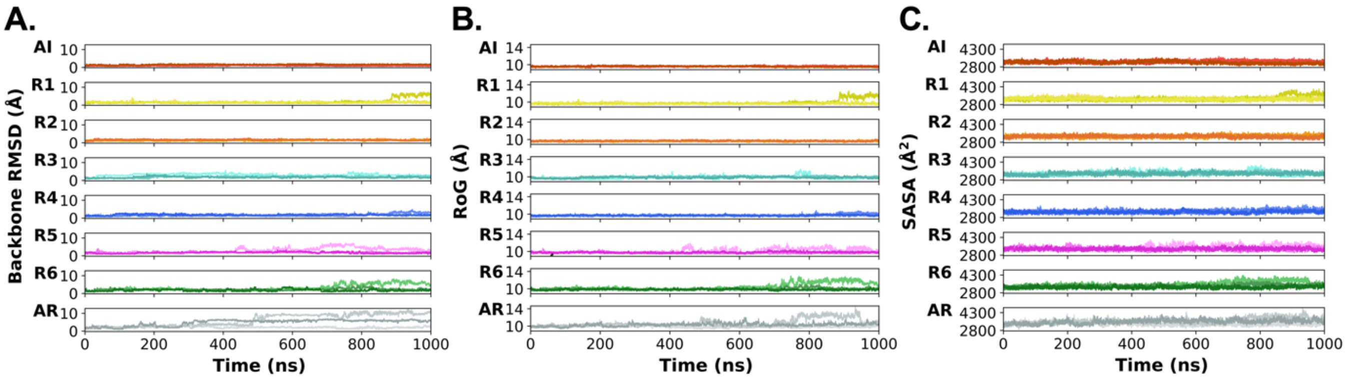

Figure S1: Timeline evolution of (A) backbone RMSD from the crystal structure of the TFF domain in Å, (B) RoG in Å, and (C) SASA in Å^2 of the TFF domain. Three individual trajectories of 1 μs are shown for fully oxidized (AI), mono- (R1-3) and di-reduced (R4-6), and fully reduced (AR) states.

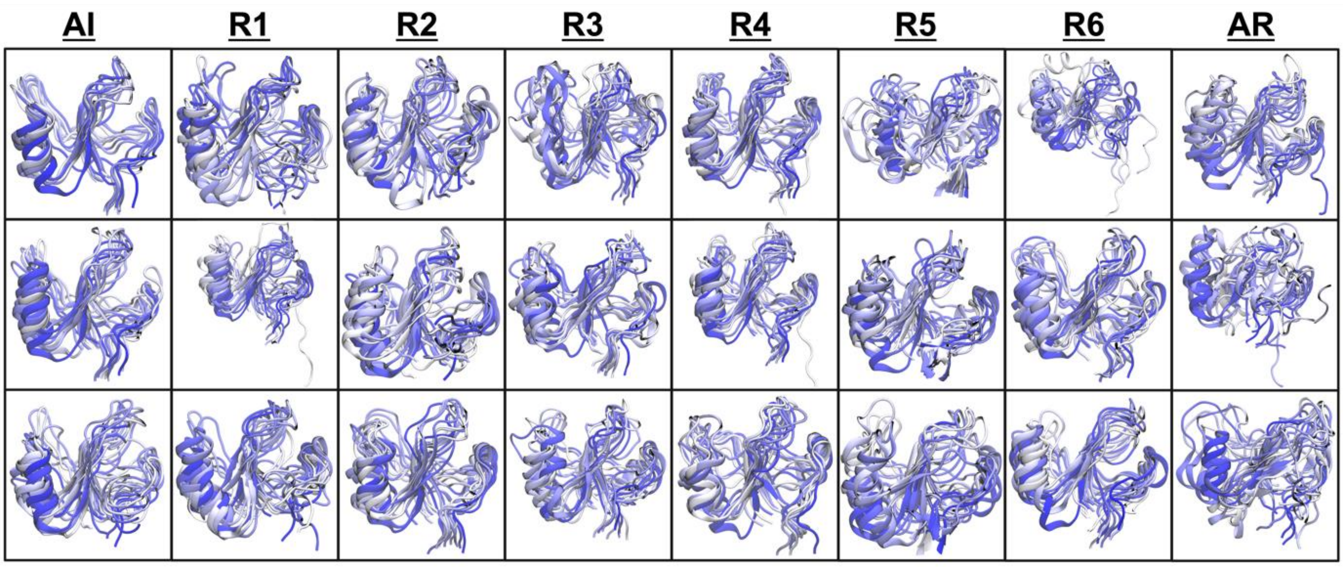

Figure S2: Dynamics of the TFF domain during MD simulations. Each column represents a different redox state from AI to AR. The boxes below belong to three independent MD simulations of 1 μs each. The domain is colored in shades of dark blue to light blue, illustrating the time-evolution of structural dynamics.

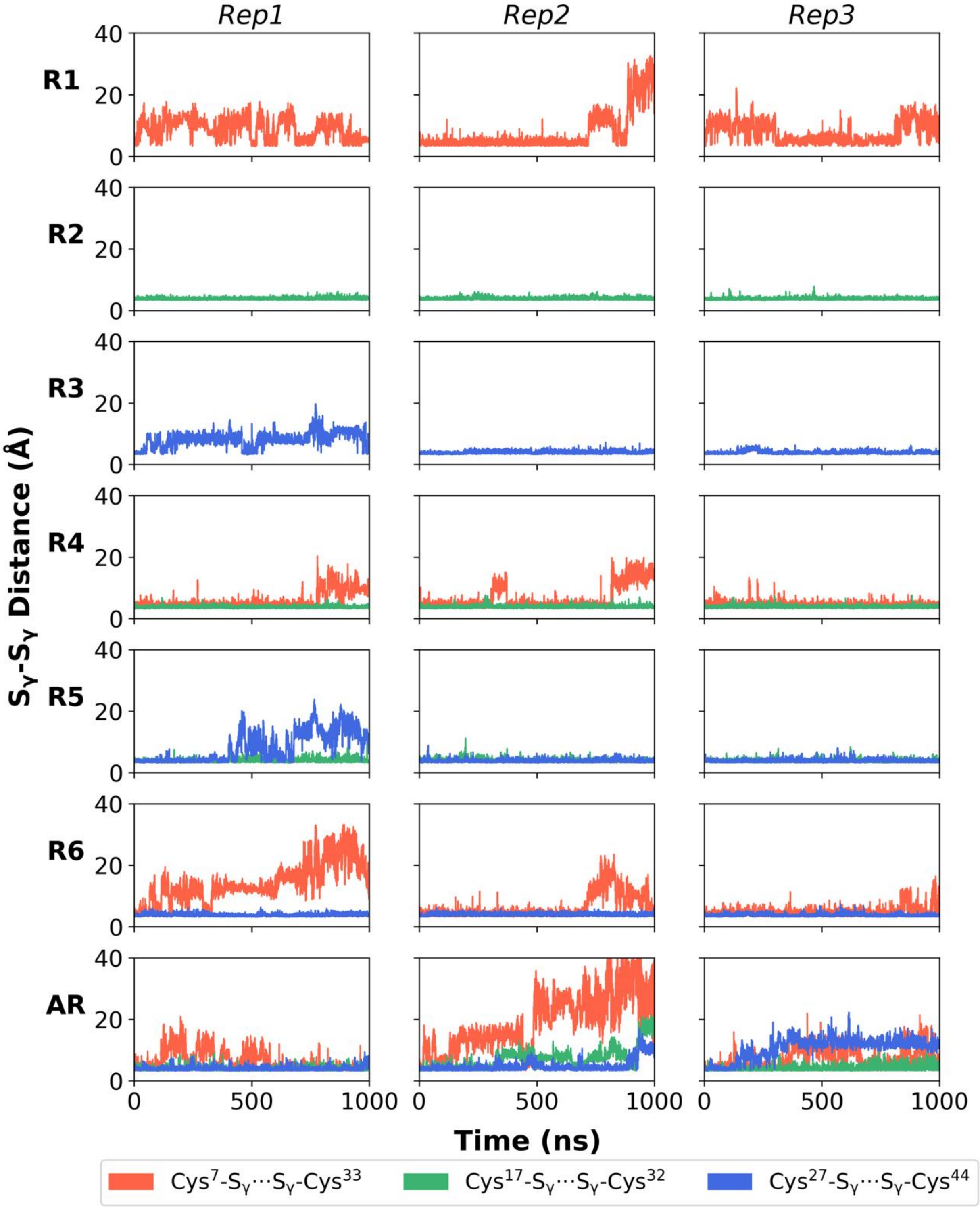

Figure S3: Time evolution of Sγ···Sγ distances in Å for reduced disulfide bonds in TFF1.

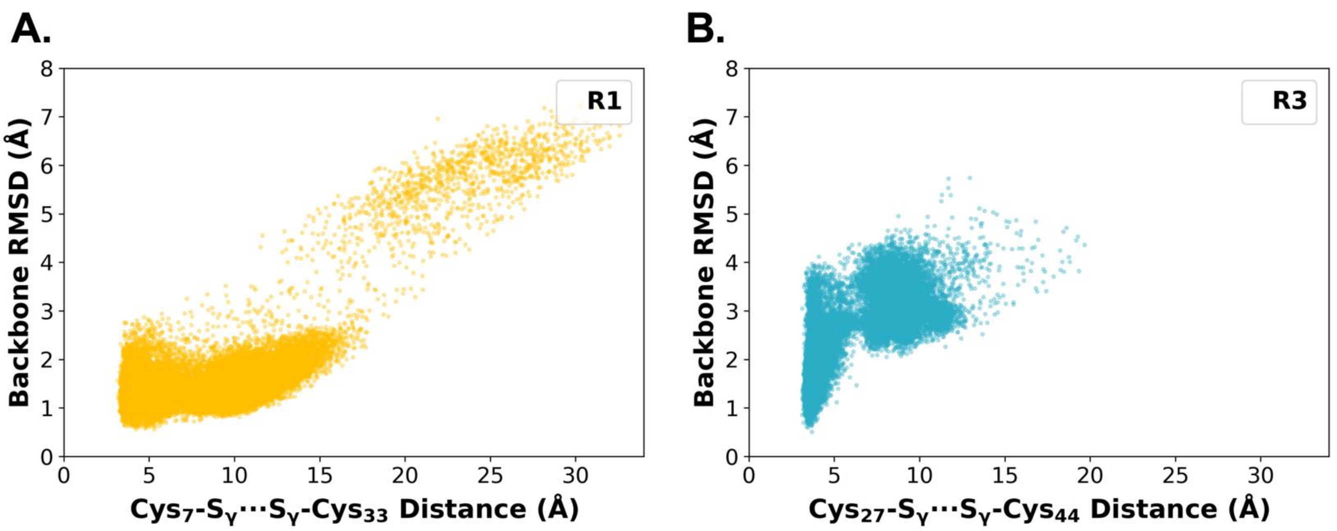

Figure S4: 2D joint scatter plots as a function of Sγ-Sγ distances of the reduced bonds and the backbone RMSD of the domain for the (A) R1 and (B) R3 states.

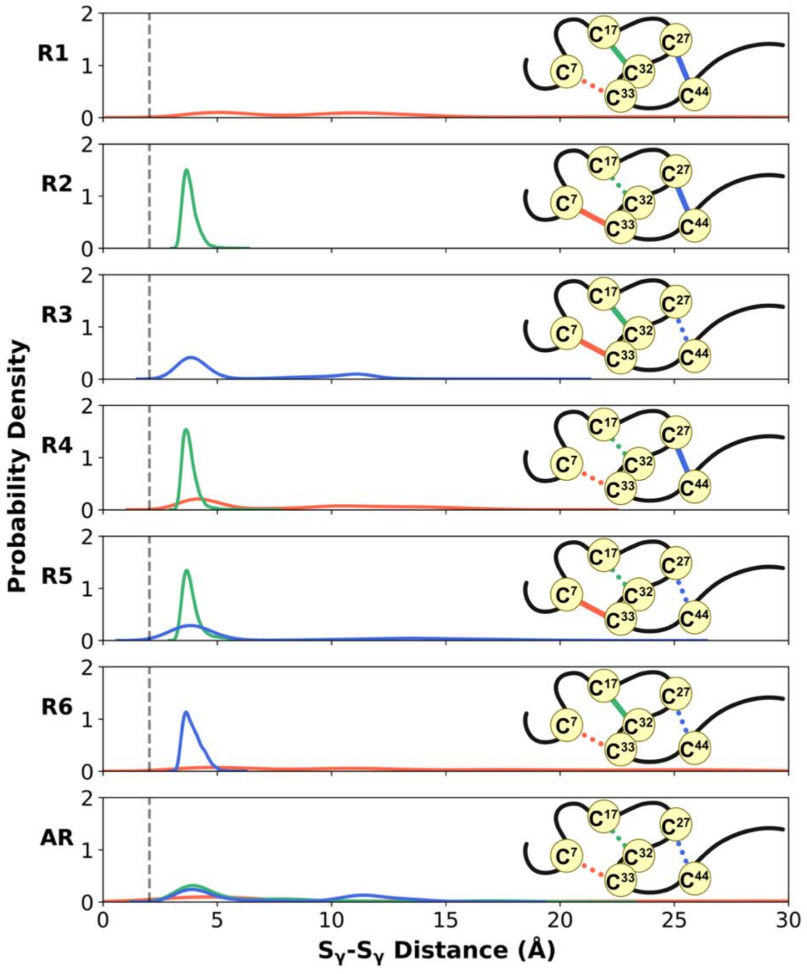

Figure S5: Probability density distributions of Sγ-Sγ distances upon the sequential reduction of disulfide bonds, Cys^{7}-Sγ···Sγ-Cys^{33} (red), Cys^{17}-Sγ···Sγ-Cys^{32} (green), and Cys^{27}-Sγ···Sγ-Cys^{44} (blue) in TFF1. Analyses was performed over the last 250 ns of each trajectory for each redox state.

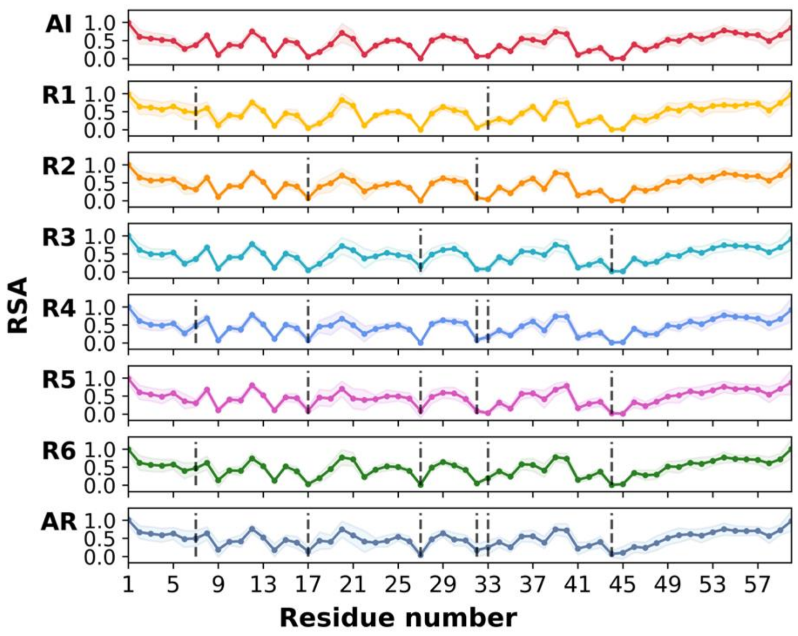

Figure S6: Relative solvent accessibility (RSA) of each residue in the TFF1 peptide. The data points are the average RSA values calculated from the concatenated trajectories of each state. Shaded regions represent the standard deviation per residue. Vertical grey dashed lines indicate the position of cysteine residues with reduced disulfide bonds. RSA values range from 0 to 1, where 0 is completely buried and 1 is fully exposed. Values between 0 and 1 correspond to moderate degrees of burial. The individual RSA values of cysteine residues can be seen in **Table S1** below.

Table S1: Average relative solvent accessibility (RSA) of cysteine residues in TFF1 across different redox states. Filled fields in gray indicate the reduced disulfide bonds in each state. Errors are represented as standard deviation.

		AI	R1	R2	R3	R4	R5	R6	AR
DSI	**Cys7**	0.37 ± 0.09	0.47 ± 0.21	0.31 ± 0.11	0.35 ± 0.09	0.48 ± 0.11	0.30 ± 0.11	0.47 ± 0.16	0.50 ± 0.21
	**Cys33**	0.07 ± 0.04	0.19 ± 0.11	0.04 ± 0.03	0.08 ± 0.07	0.17 ± 0.08	0.03 ± 0.03	0.20 ± 0.10	0.25 ± 0.19
DSII	**Cys17**	0.05 ± 0.04	0.04 ± 0.04	0.07 ± 0.06	0.05 ± 0.05	0.08 ± 0.06	0.09 ± 0.07	0.03 ± 0.04	0.14 ± 0.13
	**Cys32**	0.06 ± 0.03	0.05 ± 0.03	0.09 ± 0.04	0.08 ± 0.04	0.09 ± 0.04	0.09 ± 0.07	0.06 ± 0.03	0.17 ± 0.18
DSIII	**Cys27**	0.00 ± 0.01	0.00 ± 0.01	0.01 ± 0.01	0.15 ± 0.23	0.01 ± 0.01	0.10 ± 0.20	0.02 ± 0.03	0.04 ± 0.06
	**Cys44**	0.00 ± 0.01	0.00 ± 0.01	0.01 ± 0.02	0.02 ± 0.03	0.01 ± 0.02	0.03 ± 0.04	0.01 ± 0.02	0.07 ± 0.09
Outside of domain	**Cys58**	0.48 ± 0.23	0.53 ± 0.20	0.55 ± 0.21	0.55 ± 0.19	0.55 ± 0.20	0.54 ± 0.22	0.61 ± 0.18	0.57 ± 0.20

Table S2: Propensities of secondary structural elements —helix-1, helix-2, beta-1, and beta-2— to adopt different structural types, including alpha-helix (H), 3-10 helix (G), extended configuration (E), isolated bridge (B), turn (T), and coil (C), are given as percentages for each redox state.

	AI	R1	R2	R3	R4	R5	R6	AR
Helix-1	**H:** 0%<br>**G:** 28.3%<br>**E:** 0%<br>**B:** 0%<br>**T:** 71.7%<br>**C:** 0%	**H:** 0%<br>**G:** 26.9%<br>**E:** 0%<br>**B:** 0%<br>**T:** 68.8%<br>**C:** 4.3%	**H:** 0%<br>**G:** 20.7%<br>**E:** 0%<br>**B:** 0%<br>**T:** 79.3%<br>**C:** 0%	**H:** 0%<br>**G:** 27.2%<br>**E:** 0%<br>**B:** 0%<br>**T:** 72.8%<br>**C:** 0%	**H:** 0%<br>**G:** 22.1%<br>**E:** 0%<br>**B:** 0%<br>**T:** 77.9%<br>**C:** 0%	**H:** 0%<br>**G:** 24.9%<br>**E:** 0%<br>**B:** 0%<br>**T:** 75.1%<br>**C:** 0%	**H:** 0%<br>**G:** 23.2%<br>**E:** 0.1%<br>**B:** 0.2%<br>**T:** 68.2%<br>**C:** 8.3%	**H:** 0%<br>**G:** 19.6%<br>**E:** 0%<br>**B:** 0%<br>**T:** 74.1%<br>**C:** 6.3%
Helix-2	**H:** 98.3%<br>**G:** 0.2%<br>**E:** 0%<br>**B:** 0%<br>**T:** 0.1%<br>**C:** 1.4%	**H:** 97.8%<br>**G:** 0.2%<br>**E:** 0%<br>**B:** 0%<br>**T:** 0.2%<br>**C:** 1.8%	**H:** 98.3%<br>**G:** 0.3%<br>**E:** 0%<br>**B:** 0%<br>**T:** 0.4%<br>**C:** 1.0%	**H:** 66.5%<br>**G:** 0.9%<br>**E:** 0.4%<br>**B:** 0.9%<br>**T:** 13.9%<br>**C:** 17.4%	**H:** 97.7%<br>**G:** 0.5%<br>**E:** 0%<br>**B:** 0%<br>**T:** 0.5%<br>**C:** 1.3%	**H:** 78.6%<br>**G:** 1.8%<br>**E:** 0%<br>**B:** 0.1%<br>**T:** 10.0%<br>**C:** 9.5%	**H:** 97.8%<br>**G:** 0.6%<br>**E:** 0%<br>**B:** 0%<br>**T:** 0.6%<br>**C:** 1.0%	**H:** 72.8%<br>**G:** 5.8%<br>**E:** 0%<br>**B:** 0%<br>**T:** 16.9%<br>**C:** 4.5%
Beta-1	**H:** 0%<br>**G:** 0%<br>**E:** 98.9%<br>**B:** 0.6%<br>**T:** 0%<br>**C:** 0.5%	**H:** 0%<br>**G:** 0%<br>**E:** 99.3%<br>**B:** 0.4%<br>**T:** 0%<br>**C:** 0.3%	**H:** 0%<br>**G:** 0%<br>**E:** 95.9%<br>**B:** 2.0%<br>**T:** 0%<br>**C:** 2.1%	**H:** 0%<br>**G:** 0%<br>**E:** 97.4%<br>**B:** 1.3%<br>**T:** 0%<br>**C:** 1.3%	**H:** 0%<br>**G:** 0%<br>**E:** 94.5%<br>**B:** 2.7%<br>**T:** 0%<br>**C:** 2.8%	**H:** 0%<br>**G:** 0%<br>**E:** 96.8%<br>**B:** 1.6%<br>**T:** 0%<br>**C:** 1.6%	**H:** 0%<br>**G:** 0%<br>**E:** 90.8%<br>**B:** 4.6%<br>**T:** 0%<br>**C:** 4.6%	**H:** 1.0%<br>**G:** 0.1%<br>**E:** 79.8%<br>**B:** 5.3%<br>**T:** 0.7%<br>**C:** 13.1%
Beta-2	**H:** 0%<br>**G:** 0%<br>**E:** 99.0%<br>**B:** 0.8%<br>**T:** 0.2%<br>**C:** 0%	**H:** 0%<br>**G:** 0%<br>**E:** 99.4%<br>**B:** 0.5%<br>**T:** 0.1%<br>**C:** 0%	**H:** 0%<br>**G:** 0%<br>**E:** 96.5%<br>**B:** 3.0%<br>**T:** 0.5%<br>**C:** 0%	**H:** 0%<br>**G:** 0%<br>**E:** 98.0%<br>**B:** 1.4%<br>**T:** 0.6%<br>**C:** 0%	**H:** 0%<br>**G:** 0%<br>**E:** 96.2%<br>**B:** 3.5%<br>**T:** 0.2%<br>**C:** 0.1%	**H:** 0%<br>**G:** 0%<br>**E:** 97.7%<br>**B:** 1.8%<br>**T:** 0.4%<br>**C:** 0.1%	**H:** 0%<br>**G:** 0%<br>**E:** 93.2%<br>**B:** 3.3%<br>**T:** 3.5%<br>**C:** 0%	**H:** 0%<br>**G:** 0%<br>**E:** 80.9%<br>**B:** 8.2%<br>**T:** 3.0%<br>**C:** 7.9%

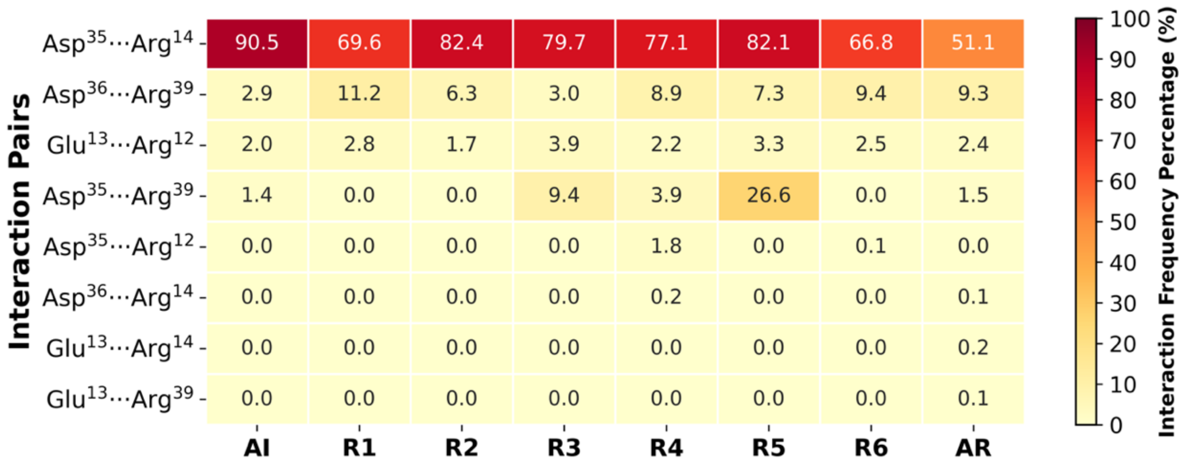

Figure S7: Frequency of possible salt bridges in the TFF domain across different disulfide bond states. Frequency percentages of interaction pairs are plotted as a heatmap, where the percentage increases from light yellow to dark red.

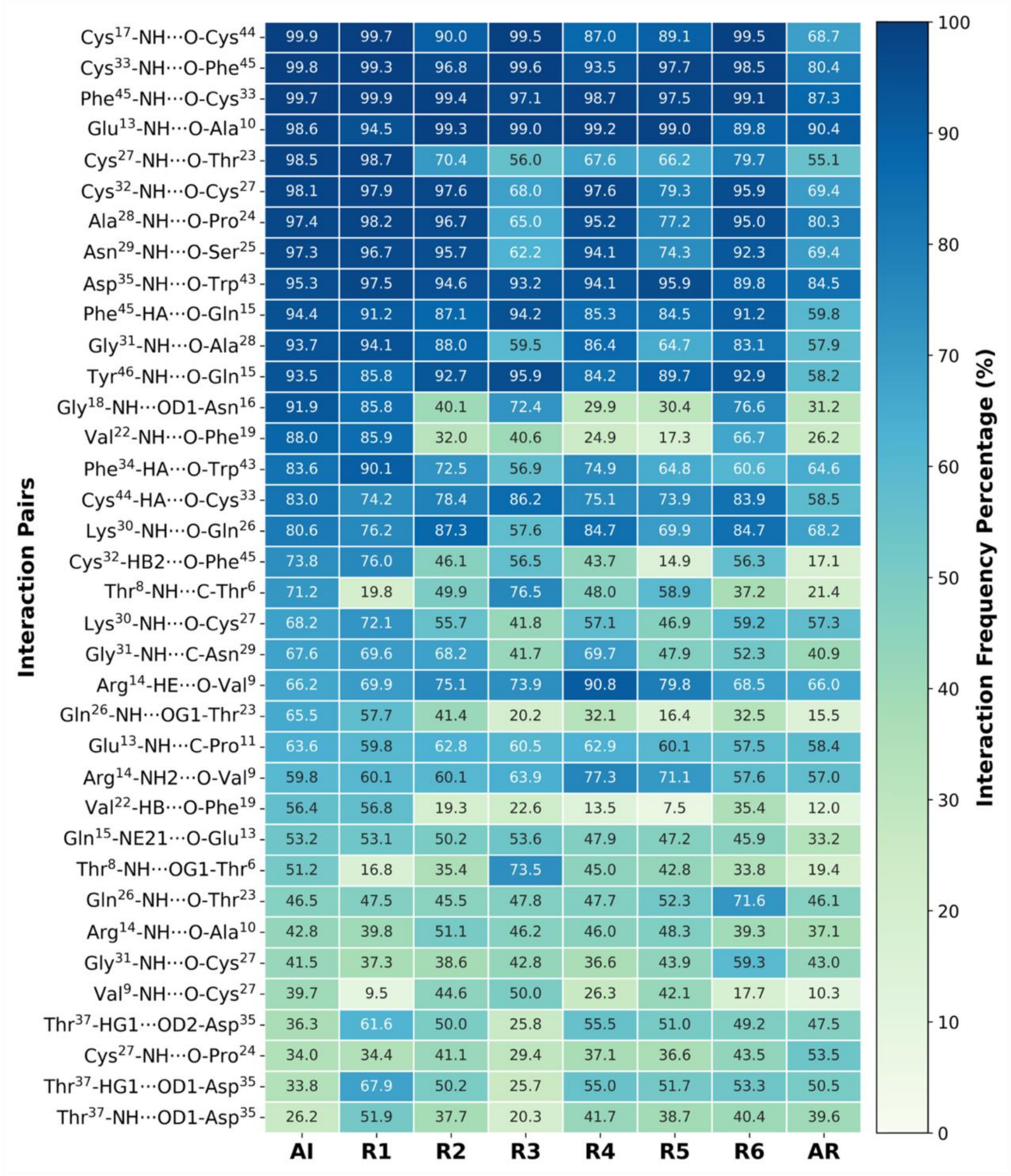

Figure S8: Hydrogen bond occupancies of interaction pairs in the TFF domain. Frequency percentages of interaction pairs are represented in a heatmap, with the percentage increments indicated by a gradient from light yellow to dark turquoise. Only interaction pairs with a minimum of 50% hydrogen bond occupancy in any of the redox states are displayed.
